# Comparison of Traditional and AI-Based Methods: Barrett Universal II vs. Ladas Super Formula in IOL Power Calculation

**DOI:** 10.3390/jcm14062023

**Published:** 2025-03-17

**Authors:** Ionela-Iasmina Yasar, Servet Yasar, Leila Al Barri, Nadina Mercea, Mihnea Munteanu, Horia Tudor Stanca

**Affiliations:** 1 Ophthalmology Department, “Victor Babes” University of Medicine and Pharmacy Timisoara, 300041 Timisoara, Romania; ionela.yasar@umft.ro (I.-I.Y.); leila.albarri@umft.ro (L.A.B.); nadina.mercea@umft.ro (N.M.); 2 Munteanu Ophthalmologic Center Timisoara, 300092 Timisoara, Romania; drservetyasar@gmail.com; 3 Oftalmo Sensory-Tumor Research Center—ORL (EYE-ENT), Ophthalmology Department, “Victor Babes” University of Medicine and Pharmacy, 300041 Timisoara, Romania; 4 Ophthalmology Department, “Carol Davila” University of Medicine and Pharmacy, 050474 Bucharest, Romania; horia.stanca@umfcd.ro

**Keywords:** intraocular lens power calculation, ARGOS, Barrett Universal II and Ladas Super Formulas, spherical equivalent

## Abstract

**Background:** Pursuing optimal visual outcomes following cataract surgery remains a cornerstone of modern ophthalmology. Central to this objective is the precise calculation of intraocular lens power. However, despite significant advancements in biometric measurements and computational algorithms, variability in refractive outcomes continues to pose a challenge. This study aims to analyze the outcomes comprehensively by reviewing established and newer techniques. **Methods:** The eyes included in this study were evaluated based on various criteria, and a total of 210 eyes which met these criteria were included in the research. Our study is a retrospectively designed observational research study. The study included individuals who had experienced successful IOL implantation to correct refractive errors or cataracts. The ARGOS SS-OCT device, a spectral-domain optical coherence tomography system, was used in this study. In measuring the lens power, values were obtained using the Barrett Universal II and Ladas Super Formulas. These values were compared. Postoperative assessments were conducted at 1–3 months and 3–12 months, including spherical equivalents. **Results:** The mean age of the participants was 63.44 ± 11.62 years. The study’s two most frequently used lens brands were ALCON and ZEISS. The lens powers calculated using the Barrett Universal II and Ladas Super Formulas were compared. The mean values calculated using both formulas were highly similar, with no statistically significant differences observed. We compared the spherical equivalent values calculated during the participants’ first and second postoperative follow-ups. The spherical equivalent values were similar, with no statistically significant differences. **Conclusions:** Formulas represent significant advances in ophthalmology and significantly improve visual outcomes; however, differences in their methodology and predictive accuracy warrant further analysis.

## 1. Introduction

Pursuing optimal visual outcomes following cataract surgery remains a cornerstone of modern ophthalmology [1]. Central to this objective is the precise calculation of intraocular lens (IOL) power, which is instrumental in determining the refractive outcome post-surgery. Given the growing demand for visual restoration with minimal dependence on corrective eyewear, particularly in advancing age-related cataract and refractive surgeries, achieving high accuracy in IOL power prediction is paramount [2]. However, despite significant advancements in biometric measurements and computational algorithms, variability in refractive outcomes continues to pose a challenge, underscoring the complexity of IOL power calculations [3].

The accuracy of IOL power predictions is influenced by many factors, including, but not limited to, corneal curvature, axial length (AL), anterior chamber depth (ACD), and the selection of the IOL formula itself. Subject to biological and measurement-related uncertainties, these parameters can significantly impact the refractive outcomes achieved postoperatively [4]. Numerous studies have sought to refine and validate the various IOL power calculation formulas used [5,6,7]. However, discrepancies between predicted and actual postoperative refraction persist, indicating the need for continued innovation and scrutiny in this field [8,9].

ARGOS^®^ (Movu, a Santec company, Milton Keynes, UK) utilizes Swept-Source Optical Coherence Tomography (SS-OCT) technology and is used to measure the key parameters of the eye, including the AL, prior to cataract surgery. This allows AL calculations to be adjusted according to the variability in the length of each segment. ARGOS is pre-programmed with the Barrett Universal II (BU II) formula, which is an updated version of the BU I formula [10].

The original Ladas Super Formula (LSF) 1.0 incorporated an axial length adjustment along the Wang–Koch axis and was composed of elements derived from the Hoffer Q, Holladay 1, and SRK/T formulas. It has been demonstrated that employing multiple formulas to select the most accurate one for a specific eye improves outcomes [11].

The LSF v1.0b builds upon the framework of version 1.0. It integrates AI-assisted learning into the calculations to predict refractive errors more accurately. These calculations are based on three input variables: the AL, corneal power, and ACD [12].

This study aims to analyze the results of these formulas comprehensively by reviewing established and newer techniques. It will contribute to the ongoing discourse on optimizing refractive prediction models, which is central to achieving superior visual outcomes for patients undergoing refractive lens exchange procedures or cataract surgeries.

## 2. Materials and Methods

### 2.1. Study Population

The eyes included in this study were evaluated based on various criteria, and a total of 210 eyes which met these criteria were included in the research.

### 2.2. Power Analysis

The sample size for this study was determined through a power analysis. With an effect size of d = 0.75, power (1 − β) = 0.90, and an allocation ratio of 1, the minimum sample size was calculated to be equal in each group, with a minimum of 93 eyes required.

### 2.3. Study Design and Participants

Our study is a retrospectively designed observational research study. The refractive outcomes achieved following successful surgeries were analyzed within the scope of this investigation. This study was conducted between July 2021 and June 2024 in the Department of Ophthalmology at the Victor Babes University of Medicine and Pharmacy in Timisoara, Romania. The study covers the data collected during this period. All participants underwent a thorough ophthalmological examination before their surgical procedure. This study includes individuals who experienced successful IOL implantation to correct refractive errors or cataracts. The same individual conducted all measurements within a single session. Three measurements were taken, and their average was used for analysis. In measuring the lens power, values were obtained using LSF and BU II formula. These values were compared. Postoperative assessments were conducted at 1–3 months and 3–12 months, including assessments of the spherical equivalent (SE). The frequencies of the eyes being within ±1.00 diopters and ±0.50 diopters were also calculated during the evaluation of SE values. Participants who did not return for follow-up at these dates were excluded from this study.

### 2.4. Exclusion Criteria

- History of ocular trauma;
- Previous ocular surgeries;
- AL measurements <21 mm or >26 mm;
- Corneal or vitreous opacities;
- Dry eye syndrome;
- Retinal pathologies;
- Glaucoma or nystagmus;
- Not returning for follow-up.

### 2.5. Surgical Procedure

The surgical procedure performed on the eyes involved a main incision made at an angle of 110 degrees and with an incision width of 2.2 mm. The first and second side incisions were created at angles of 30 degrees and 160 degrees, respectively, with each having an incision width of 1.2 mm. This study utilized monofocal, EDOF (Extended Depth of Focus), and trifocal lenses. The most commonly used brands were Alcon (Alcon Laboratories, Inc., Fort Worth, TX, USA), ZEISS (Carl Zeiss Meditec, Jena, Germany), and Johnson & Johnson (Basel, Switzerland).

### 2.6. ARGOS

The ARGOS SS-OCT device is a spectral-domain optical coherence tomography system that operates at a wavelength of 1060 nm, with a bandwidth of 20 nm and an A-scan rate of 3000 scans per second. It provides two-dimensional OCT imaging capabilities. Keratometry (K) is measured using a ring of 16 LEDs with a diameter of 2.2 mm. The device employs a corneal refractive index of 1.3375. The corneal diameter is measured based on the OCT image, which is further utilized as a reference to calculate the white-to-white (WTW) value under the guidance of Alcon’s imaging system. Central corneal thickness (CCT), ACD, Lens Thickness (LT), and AL are measured using OCT, taking into account the distinct refractive indices of each medium: the cornea (1.376), aqueous and vitreous humor (1.336), and lens (1.410).

### 2.7. Examined Variables

- Gender;
- Age of the participants;
- AL;
- ACD;
- LT;
- WTW;
- K (flat and steep);
- SE.

### 2.8. Groups

- Group 1: The BU II formula. The most up-to-date version registered in the ARGOS device was used on the scheduled date of surgery.
- Group 2: The LSF used in this study was from the latest version of the website accessed [13].

### 2.9. Ethics

The ethical approval for this study was obtained from the Ethics Committee of the Victor Babes University of Medicine and Pharmacy in Timisoara, Romania. All stages of the study were conducted in accordance with the principles outlined in the Declaration of Helsinki.

### 2.10. Statistical Analysis

Statistical analyses were conducted using SPSS version 25 (IBM Corp., Armonk, NY, USA). Descriptive statistics have been presented, including the count, percentage, mean ± standard deviation, median, minimum, and maximum values. The normality of the data was assessed using the Kolmogorov–Smirnov test. Statistical relationships between group means were analyzed through Student’s *t*-test. Potential correlations were examined using Pearson correlation analysis. A *p*-value of less than 0.05 was considered the threshold for statistical significance.

## 3. Results

In our study, 210 eyes were examined. In total, 126 of the participants were female (60%) and 84 were male (40%). The mean age of the participants was 63.44 ± 11.62 years. The youngest participant was 39, while the oldest was 91. The demographic data of the participants and the measurement results obtained from ARGOS are presented in Table 1.

This study’s two most frequently used lens brands were ALCON and ZEISS. The subtypes of ALCON lenses used included monofocal, EDOF, and trifocal lenses, of which monofocal lenses were the most commonly used. As for the ZEISS lenses, monofocal and multifocal subtypes were employed, with monofocal lenses being more frequently utilized compared to multifocal lenses (Table 2).

The lens powers calculated using the BU II and LSFs were compared. The means of the two calculations were found to be very close, and no statistically significant difference was observed between them (*p* = 0.3) (Table 3).

A potential correlation between the BU II and LSFs was analyzed using Pearson correlation analysis. The results showed that there was no statistically significant correlation between the data obtained from the two formulas (*p* = 0.5) (Table 4).

We compared the SE values calculated during the participants’ first and second postoperative follow-ups. The SE values were similar, with no statistically significant difference seen (*p* = 0.6) (Table 5).

**Figure 1:**
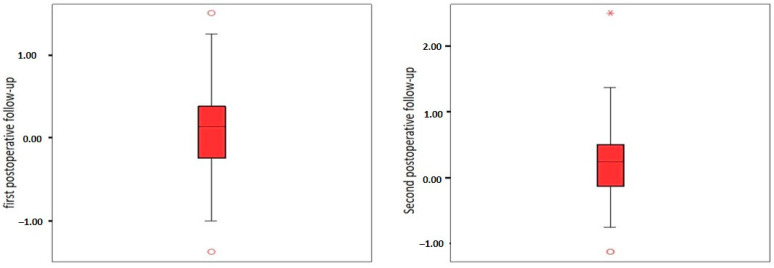
Boxplot graphic of SE values. Minimum, first (25%) quartile median, third (75%) quartile, and maximum value are shown. * The extreme outlier.

The analysis of the calculated SE values for both controls revealed that the frequencies of eyes falling within the ±1.00 and ±0.50 diopters were similar (Table 6, Figure 1).

## 4. Discussion

Our study aimed to compare more traditional techniques with newer methods. Within this scope, the outcomes associated with these techniques have been examined through comprehensive analyses. It is anticipated that the findings will contribute to the optimization of the refractive prediction models used to achieve superior outcomes for patients undergoing refractive lens exchange and cataract surgery.

Both formulas represent significant advances in ophthalmology and significantly improve visual outcomes after cataract surgery. However, the significant differences in their methodology and predictive accuracy deserve further discussion. BU II has a solid theoretical basis. It incorporates the concept of effective lens position and uses a five-variable matrix to optimize IOL power estimation. This formula has shown superior accuracy, especially in eyes with extreme biometric features. This is due to its ability to integrate ACD and K measurements with the axial length, thus minimizing errors in postoperative refractive outcomes. Consistent with the literature, BU II exhibited a lower mean absolute error (MAE) than traditional third- and fourth-generation formulas, underscoring its reliability and predictive accuracy. BU II demonstrated a superior predictive accuracy in extremely long eyes [14].

The LSF represents a paradigm shift, as it utilizes machine learning algorithms to adapt to biometric differences. The LSF offers a personalized approach to IOL power calculation by integrating experimental data from extensive databases. Since the LSF tailors predictions to individual eyes, it lacks the limitations of traditional theoretical formulas. It is particularly promising in managing complex cases such as eyes with irregular corneal shapes or refractive surgery. The LSF approach exhibited more significant variability in its predictions. These findings reinforce our observation that the LSF systematically generates higher IOL power values, which may introduce a predisposition toward hyperopic outcomes if not interpreted cautiously [11].

The findings of several studies provide valuable insights into the topic. In one study, Pereira et al. demonstrated that the Kane formula provided more accurate data than the BU II formula and LSF [15]. Similarly, another study revealed that the Kane formula outperformed next-generation formulas in terms of accuracy [16]. Darcy et al. conducted a study with a huge sample size, concluding that while BU II and LSF yielded highly reliable results, they were inferior to the specific other formulas with which they were compared [17].

In another study, BU II and LSF were also found to produce reliable results; however, the Kane formula again demonstrated a superior performance compared to the other formulas analyzed. Partial coherence interferometry measurements were employed in that study [18]. Conversely, Rocha-de-Lossada et al. found that the Emmetropia Verifying Optical Formula (EVO) yielded lower MAE values when compared with the other formulas evaluated in their study [19].

In another study supporting the superiority of BU II, Khatib et al. reported that BU II produced the lowest MAE values when compared to EVO, Hill-RBF, LSF, and several other formulas [20]. Similarly, Nemeth et al., in a study comparable to the present research, highlighted the prominence of BU II by comparing it with AI-based intraocular lens (IOL) formulas. The AI-based IOL formulas included in their study were Hill-RBF 2.0, Kane, and PEARL-DGS. While LSF was not included in their comparisons, their findings provide valuable insights, showing that BU II performed better than the other formulas evaluated [21].

The observed variability in results across studies is attributed to differences in the types of lenses analyzed and the variations in sample sizes. Some studies included over ten thousand participants [17], while others evaluated two monofocal IOL lenses instead of a single lens [21]. These methodological differences likely explain the discrepancies in the reported findings.

In a study conducted in Korea and performed on more than 1500 eyes, the newly developed Eom IOL power calculator formula was compared with other formulas including BU II and LSF. The results showed that EOm provided more accurate results than the other formulas. The next most reliable results were determined to be those provided by BU II and LSF. In comparisons made in terms of the MedAE and IOL Formula Performance Indexes, it was observed that LSF provided more accurate results than BU II [22]. These results are consistent with those obtained in our study.

When comparing these formulas, it is important to consider their performance in specific clinical scenarios. It has been noted that BU II performs better regarding refractive predictability in eyes with extreme axial lengths. This can be attributed to its high predictive power in terms of posterior corneal curvature [23]. The LSF, on the other hand, can handle complex biometric variations better than traditional formulas, thanks to the adaptability provided by machine learning. Thus, it offers an innovative approach to personalized medicine [11]. Another difference between the formulas is the ease of clinical application. BU II guarantees worldwide accessibility. It is integrated into commercially available biometry devices [24]. In contrast, the LSF requires special software or computational resources to implement [25]. This is a limitation in resource-constrained environments.

There are some limitations in our study. First, the number of participants is likely to be limited. Increasing the sample size is extremely important for ensuring the generalizability of the data obtained. Second, only two formulas were examined in our study. Our study is a pilot study, and studies comparing more formulas will be conducted in the future. The design of our study is another limitation. Prospective studies provide more reliable results than retrospective studies. The control of exposure factors is limited in retrospective studies. Missing history/background and/or missing information are a shared limitation in both. Selection and recall bias may affect the accuracy of the results. These biases arise when participants do not represent the general population or when there are inconsistencies in data recollection or reporting. Variations in the types of lenses analyzed and differences in sample sizes may lead to variability in the results. This variability can complicate the interpretation of findings and raise doubts regarding their clinical applicability.

## 5. Conclusions

The lens power averages of the BU II formula and LSF were almost the same, indicating that both formulas provide comparable prediction accuracy. The results obtained from our Pearson correlation analysis also showed that there was no significant correlation between the data obtained from the two formulas, indicating that neither formula is superior to the other.

In addition, the SE values recorded at the first and second postoperative follow-ups confirmed the consistency of the formulas. This finding emphasizes the clinical reliability of both formulas in providing stable postoperative refractive outcomes. According to these results, neither the traditional nor the AI-based method has a significant advantage in terms of accuracy or prediction consistency.

In this context, in clinical practice, the choice of one formula may be guided by surgeon preference, ease of integration into the workflow, or specific patient characteristics, rather than intrinsic methodological superiority. AI is considered a revolution in ophthalmic calculations. Our study emphasized the importance of rigorous validation before adopting AI-based formulas as definitive replacements for traditional methods. Studies conducted on larger samples and with different patient demographics may provide more information on the performance of these formulas in various clinical scenarios.

## Figures and Tables

**Table 1:** Descriptive statistics.

	*n*	Min	Max	Mean ± SD
Gender	210			
Age		39	91	63.44 ± 11.62
AL		21.13	25.77	23.2818 ± 0.83
K1		39.99	48.33	43.5660 ± 1.45
K2		40.50	48.89	44.2356 ± 1.52
ACD		2.52	4.11	3.2176 ± 0.33
LT		3.45	5.53	4.5334 ± 0.37
WTW		10.80	12.93	11.9417 ± 0.37

**Table 2:** Descriptive statistics of the two most commonly used lens brands and their subtypes, classified by focal points.

	*n*	%
ALCON *	129	61.1
Monofocal **	58	44.9
EDOF **	23	17.8
Trifocal **	48	37.3
ZEISS *	44	20.8
Monofocal **	28	36.6
Multifocal **	16	63.4

* The frequency distribution of all brands utilized in this study. ** The frequency distribution within the ALCON/ZEISS brand.

**Table 3:** The relationship between the BU II formula- and LSF-calculated lens powers.

	*n*	Mean ± SD	Std. Error	t	df	*p*
BU II	210	21.68 ± 2.11	0.14	−0.85	418	0.3
LSF	210	21.85 ± 2.12	0.14			

**Table 4:** Correlation analysis between BU II and LSFs.

		BU II	LSF	*p*
BU II	Pearson Correlation	1	−0.004	0.5
	Sig. (2-tailed)		0.9	
LSF	Pearson Correlation	−0.004	1	
	Sig. (2-tailed)	0.9		

**Table 5:** Comparison of SE values (diopters) calculated during the first and second postoperative follow-ups.

	*n*	Mean ± SD	Std. Error	t	df	*p*
First postop. follow-up	210	0.13 ± 0.48	0.03	−0.4	418	0.6
Second postop. follow-up	210	0.14 ± 0.49	0.03			

**Table 6:** Frequencies of the eyes being within ±1.00 diopters and ±0.50 diopters.

	*n*	±1 D	±0.50 D
First postop. follow-up	210	%96.7	%89.6
Second postop. follow-up	210	%96.2	%89.6

## Data Availability

The data supporting the findings of this study are available from the corresponding author upon reasonable request.

## Abbreviations

AI	Artificial intelligence
IOL	Intraocular lens
AL	Axial length
ACD	Anterior chamber depth
SS-OCT	Swept-Source Optical Coherence Tomography
BU II	Barrett Universal II
LSF	Ladas Super Formula
SE	Spherical equivalent
EDOF	Extended Depth of Focus
WTW	White-to-white
CCT	Central corneal thickness
K	Keratometry
